# Swedish Medical LLM Benchmark: development and evaluation of a framework for assessing large language models in the Swedish medical domain

**DOI:** 10.3389/frai.2025.1557920

**Published:** 2025-07-11

**Authors:** Birger Moëll, Fabian Farestam, Jonas Beskow

**Affiliations:** ^1^Division of Speech Music and Hearing, KTH Royal Institute of Technology, Stockholm, Sweden; ^2^Department of Mathematics, ETH Zurich, Zürich, Switzerland

**Keywords:** healthcare AI safety, large language models (LLM), emergency medicine, general medicine, medical knowledge, Swedish language understanding, retrieval-augmented generation (RAG), open source

## Abstract

**Introduction:**

We present the Swedish Medical LLM Benchmark (SMLB), an evaluation framework for assessing large language models (LLMs) in the Swedish medical domain.

**Method:**

The SMLB addresses the lack of language-specific, clinically relevant benchmarks by incorporating four datasets: translated PubMedQA questions, Swedish Medical Exams, Emergency Medicine scenarios, and General Medicine cases.

**Result:**

Our evaluation of 18 state-of-the-art LLMs reveals GPT-4-turbo, Claude- 3.5 (October 2023), and the o3model as top performers, demonstrating a strong alignment between medical reasoning and general language understanding capabilities. Hybrid systems incorporating retrieval-augmented generation (RAG) improved accuracy for clinical knowledge questions, highlighting promising directions for safe implementation.

**Discussion:**

The SMLB provides not only an evaluation tool but also reveals fundamental insights about LLM capabilities and limitations in Swedish healthcare applications, including significant performance variations between models. By open-sourcing the benchmark, we enable transparent assessment of medical LLMs while promoting responsible development through community-driven refinement. This study emphasizes the critical need for rigorous evaluation frameworks as LLMs become increasingly integrated into clinical workflows, particularly in non-English medical contexts where linguistic and cultural specificity are paramount.

## 1 Introduction

The integration of language models (LMs) in medicine presents both significant opportunities and challenges. Large language models (LLMs) have demonstrated promising capabilities in various healthcare applications, from answering medical questions to assisting in clinical decision-making (Omiye et al., 2023; Wang Y. et al., 2023; Gianola et al., 2024). These models have the potential to enhance clinical decision-making, automate healthcare tasks, and improve patient outcomes (Li et al., 2023; Wang Y. et al., 2023). However, the risks associated with their use are substantial; inaccurate medical advice generated by these models could lead to severe consequences, including misdiagnosis and inappropriate treatment, potentially compromising patient health (Ziaei and Schmidgall, 2023; van Nuland et al., 2024). Given these risks, the integration of LLM in healthcare requires a thorough evaluation to ensure benefits and minimize risks (Wang G. et al., 2023; Barnard et al., 2023). A critical step in determining the suitability of language models for medical applications is to assess their performance using domain- and language-specific benchmarks.

Constructing a benchmark using multiple-choice questions (MCQs) is a standard way to evaluate LLM performance with many LLM evaluation benchmarks made in this format (Hendrycks et al., 2020; Zellers et al., 2019), including medical LLM benchmarks (Jin et al., 2019; Yao et al., 2024).

A benefit of MCQ benchmarks is that they can be evaluated without a human in the loop, which makes them ideal for use early in the LLM evaluation process, for instance, as part of training new models. A drawback of MCQ benchmarks is the potential for data leakage and training on test data that can artificially inflate scores but make models brittle to errors (Zhou et al., 2023; Ni et al., 2025).

Another type of LLM evaluation is human evaluation, including human preference (Chiang et al., 2024), where humans evaluate the best-performing models. Human evaluation is considered the gold standard, with the Chatbot Arena Elo Rating of LLMs seen as a de facto leaderboard of the best-performing LLM models. Still, human rating has drawbacks with human bias (Chen et al., 2024) and cost/difficulty of finding expert human raters as significant challenges. Furthermore, Chatbot Arena can be gamed, which can result in relative performance gains of up to 112% on the arena distribution (Singh et al., 2025).

A third type of evaluation is using LLM-as-a-judge, where one LLM model is used to evaluate the output of another LLM according to some criteria, similar to a human evaluation (Tan et al., 2024). The benefit of LLM-as-a-judge is the ability to rate free text responses automatically, and it does not require any gold standard response. However, studies such as Zhu et al. (2023) and Wataoka et al. (2024) show that using LLMs to evaluate responses introduces biases, such as position bias, knowledge bias, model bias, and format bias. Furthermore, the results have been shown to differ from humans even in simple setups (Thakur et al., 2024).

Although there are several MCQ benchmarks for evaluating language models in the medical domain, they focus primarily on English-language tasks and often include questions that do not fully represent real-world clinical scenarios (Omiye et al., 2023) (see Table 1 for an overview of Medical LLM benchmarks). Language-specific evaluation is important since LLMs are more capable in high resource languages, such as English (Li et al., 2025; Romanou et al., 2024), and medical practices vary between countries. Research has been published regarding non-English language evaluation of LLMs in the medical domain (Rossettini et al., 2024), including in the Swedish language (Arvidsson et al., 2024), where the focus was on comparing general practitioners' ability to the ability of LLMs. A Swedish MCQ dataset exists in the form of MedQA-SWE (Hertzberg and Lokrantz, 2024), which uses questions posed in the theoretical exam given to assess the knowledge of foreign doctors wanting to obtain a Swedish medical license. Vakili et al. (2025) focuses instead on evaluation of encoder models (Devlin et al., 2019) in the Swedish medical domain.

**Table 1:** Comparison of SMLB and Selected Medical LLM Benchmarks.

**Benchmark**	**Language**	**Question source**	**Format**	**Focus areas**	**Size**	**Access**
SMLB	Swedish	Med exams, PubMedQA (transl.), EM/GM cases	MCQ, Y/N/Maybe	Swedish med knowledge, clinical reasoning, emergency medicine, general practice	2,665	Open/code
MedQA-SWE	Swedish	Exam questions for foreign doctors	MCQ	Swedish med knowledge	3,180	Open
MedQA (USMLE)	English	USMLE exams	MCQ	Clinical knowledge, diagnosis	12.7k	Open/code
PubMedQA	English	PubMed abstracts	Y/N/Maybe, Long	Literature reasoning	1,000	Open
MedMCQA	English	Indian PG exams	MCQ	Med knowledge, clinical subjects	194k	Open/code
MMLU (Med)	English	Academic topics	MCQ	Academic/prof. med knowledge	1,871	Widely used
MultiMedQA	English	Exams, PubMed, web queries	MCQ, Free-form	Pro/consumer Q&A, safety, factuality	200k	Google research
MedQA-CS	English	Simulated OSCE	Instr.-follow	Clinical skills (notes, dx)	1,667	Open

Still, no medical benchmark consisting of multiple datasets for LLM evaluation in different medical domains exists in Swedish.

To address these limitations, we introduce the Swedish Medical LLM Benchmark (SMLB), a benchmarking suite consisting of Swedish-language questions in four distinct areas:

1. SWE-PUBMEDQA: translated PubMedQA questions.
2. SWE MEDICAL EXAMS: questions from Swedish Medical Exams.
3. SWE SPEC EM: clinical specialist questions for the emergency medicine domain.
4. SWE SPEC GM: clinical specialist questions for the general practitioner domain.

This benchmark aims to provide a more accurate and holistic assessment of LLMs' capabilities in the Swedish medical context, ensuring that the models are evaluated against tasks that closely resemble clinical practice in Sweden. With permission from the authors of MedQA-SWE, the dataset is also included in SMLB, although it has not yet been evaluated outside of the evaluation done in Hertzberg and Lokrantz (2024).

## 2 Method

Building an evaluation framework for LLMs involves structuring high-quality data into a standardized format, such as multiple-choice questions, and developing software to systematically execute the evaluation. For our framework, we chose the Python programming language since it is a common language used in the development of AI systems. The Swedish Medical LLM Benchmark (SMLB) was developed using a multifaceted approach to create a comprehensive and robust evaluation tool for large language models (LLMs) in the Swedish medical domain. Our methodology focused on three key strategies:

1. Translating existing English-language medical questions into Swedish using LLMs with manual validation.
2. Creating high-quality clinical patient cases from medical information about various disorders, assisted by LLMs.
3. Incorporating standard medical tests used to assess medical students at Swedish Medical Universities, specifically also introducing Swedish medical praxis.

### 2.1 Question format and evaluation

Our benchmarks use multiple-choice questions (MCQs) in which the LLM selects the most plausible answer from a set of options of which one is correct, in line with established practices in medical education and assessment (Case and Swanson, 2002).

### 2.2 Prompting

The primary objective of this study was to establish a standardized and unbiased benchmark, demonstrating its value independently of prompt optimization efforts. To achieve this, we deliberately minimized prompt engineering by providing simple, straightforward, and uniform prompts to all evaluated models. See Appendix A for the prompts.

This minimal prompting strategy serves two crucial purposes. First, it ensures comparability across models, preventing bias toward more widely used LLMs for which researchers typically have greater prompting experience. Second, it avoids artificially inflating scores for particular models through extensive prompt tuning.

### 2.3 Evaluation framework

The evaluation framework was written in Python by the authors B.M. and F.F. Through this framework, LLMs can be evaluated either through API access, where an API key is needed, or through local LLMs that can be run on a local device. The framework is intentionally lightweight with few external dependencies and modular, so new benchmarks can be added with ease.

Furthermore, error bars have been added using the method described in Miller (2024). The clustering method was used for SMDT due to it containing follow-up questions, placing follow-up questions in the same cluster (see Supplementary Table 7 to compare with no clustering). To create an error bar for SMLB, we used the standard error of the weighted mean of the error bars of the sub-benchmarks. This can be done since there is no question overlap between the sub-benchmarks, and there are set answers; thus, each sub-benchmark can be viewed as independent. Note that all error bars reported in the study correspond to the 95% level.

### 2.4 Open source and collaborative approach

The Swedish Medical LLM Benchmark is an open-source project. We actively encourage participants to contribute with both improvements to our benchmarking tool and by adding additional datasets.

### 2.5 Benchmarks

The Swedish Medical LLM Benchmark comprises four distinct benchmarks, each designed to evaluate different aspects of medical knowledge and reasoning (see Tables 2–4 for an overview of the benchmark).

**Table 2:** Swedish Medical LLM Benchmark.

**Benchmark**	**Description**
PubMedQA-Swedish (PQ-S)	1,000 yes/no/maybe questions; Translated from English PubMedQA (Jin et al., 2019); LLM translation with human review; allows multilingual comparison.
Medical Doctors Knowledge Test (SMDT)	535 multiple-choice questions (5 options); Adapted from Swedish clinical exams; covers various medical specialties; Assesses broad medical knowledge (Norcini et al., 2011).
Emergency Medicine (SE-EM)	464 multiple-choice questions based on patient description with follow-up questions (4 options); focuses on time-critical scenarios; Tests ability to identify and prioritize severe medical issues.
General Medicine (SE-GM)	666 multiple-choice questions based on patient description with follow-up questions (4 options); Covers 200+ common disorders; Reflects >50% of patient interactions; Tests diagnosis and severity assessment in primary care.

**Table 3:** Average question and option lengths by benchmark.

**Benchmark**	**Avg. Q chars**	**Avg. Q words**	**Avg. opt chars**	**Avg. opt words**
SE-GM	360.3	57.8	21.1	2.6
SE-EM	319.1	49.8	22.9	2.8
PQ-S	82.5	8.2	3.7	1.0
SMDT	1487.8	230.0	35.8	4.8
SMLB	475.2	72.4	17.8	2.5

**Table 4:** Composition of the Swedish Medical LLM Benchmark.

**Benchmark**	**Number of questions**	**Percentage**
PQ-S-1000	1,000	37.52%
SMDT	535	20.08%
EM	464	17.41%
GM	666	24.99%
**Total**	2,665	100.00%

#### 2.5.1 PubMedQA-Swedish-1000 (PQ-S)

PubMedQA-Swedish is a translated version of the PubMedQA dataset, including 1,000 questions with yes/no/maybe answers.

- Content: translated from the original English PubMedQA dataset (Jin et al., 2019). Utilized state-of-the-art LLMs for translation (GPT-4) (Jiao et al., 2023), followed by human review to ensure accuracy.
- Evaluation: when evaluating, each LLM is asked to answer only with “yes,” “no,” or “maybe” for each question.
- Significance: this dataset tests the model's ability to comprehend and reason about medical literature in Swedish, a crucial skill for evidence-based practice (Smith et al., 2018).

#### 2.5.2 Swedish medical doctors knowledge test (SMDT)

This dataset consists of questions from the clinical exam for doctors in Sweden, adapted for LLM evaluation.

- Content: a total of 535 multiple-choice questions, each with five different answer options.
- Rational: covers a wide range of medical specialties and topics relevant to clinical practice in Sweden. Questions related to images have been omitted to focus on text-based reasoning.
- Significance: this dataset assesses the LLM's medical knowledge across various specialties, mimicking the breadth of knowledge required of practicing physicians (Norcini et al., 2011).

#### 2.5.3 Emergency medicine (EM)

Specialist exam emergency service (EM) is a benchmark focused on time-critical healthcare issues that are in the domain of a specialist in emergency medicine.

- Content: a total of 464 multiple-choice questions, each with four different answer options. The questions cover a variety of emergency scenarios, focusing on realistic scenarios in emergency medicine.
- Rationale: including these questions was crucial as it is vital for any model in healthcare to recognize when an issue requires immediate medical attention.
- Significance: performance on these questions is a key indicator of a model's safety for general audience use, as it demonstrates the ability to identify and prioritize severe medical issues (Croskerry, 2013).

#### 2.5.4 General medicine (GM)

Specialist exam general medicine (GM) is focused on general medicine questions that are in the domain of a specialist in general medicine. General Medicine is the specialty that has the largest volume of patient interaction, and it is the benchmark for questions on common medical issues.

- Content: a total of 666 multiple-choice questions covering more than 200 common disorders encountered in general medicine.
- Rationale: encompasses more than 50% of all patient interactions, reflecting the diverse nature of primary care.
- Significance: this dataset tests the model's ability to accurately diagnose common disorders and assess their severity, mirroring the key skills required in general practice (Reilly, 2016).

The question and answer lengths of the different subbenchmarks can be seen in Table 3.

### 2.6 Evaluation metrics

We used accuracy as our evaluation metric for all our benchmarks. Furthermore, a total SMLB score was calculated, with each benchmark weighted proportionally to the number of questions included. The score was calculated with a total accuracy of 100, being a perfect score on all tests.

By combining these diverse datasets and rigorous evaluation methods, the Swedish Medical LLM Benchmark aims to provide a comprehensive assessment of LLMs' capabilities in the Swedish medical domain, focusing on both broad medical knowledge and critical decision-making skills essential for safe and effective healthcare applications.

## 3 Result

**Figure 1:**
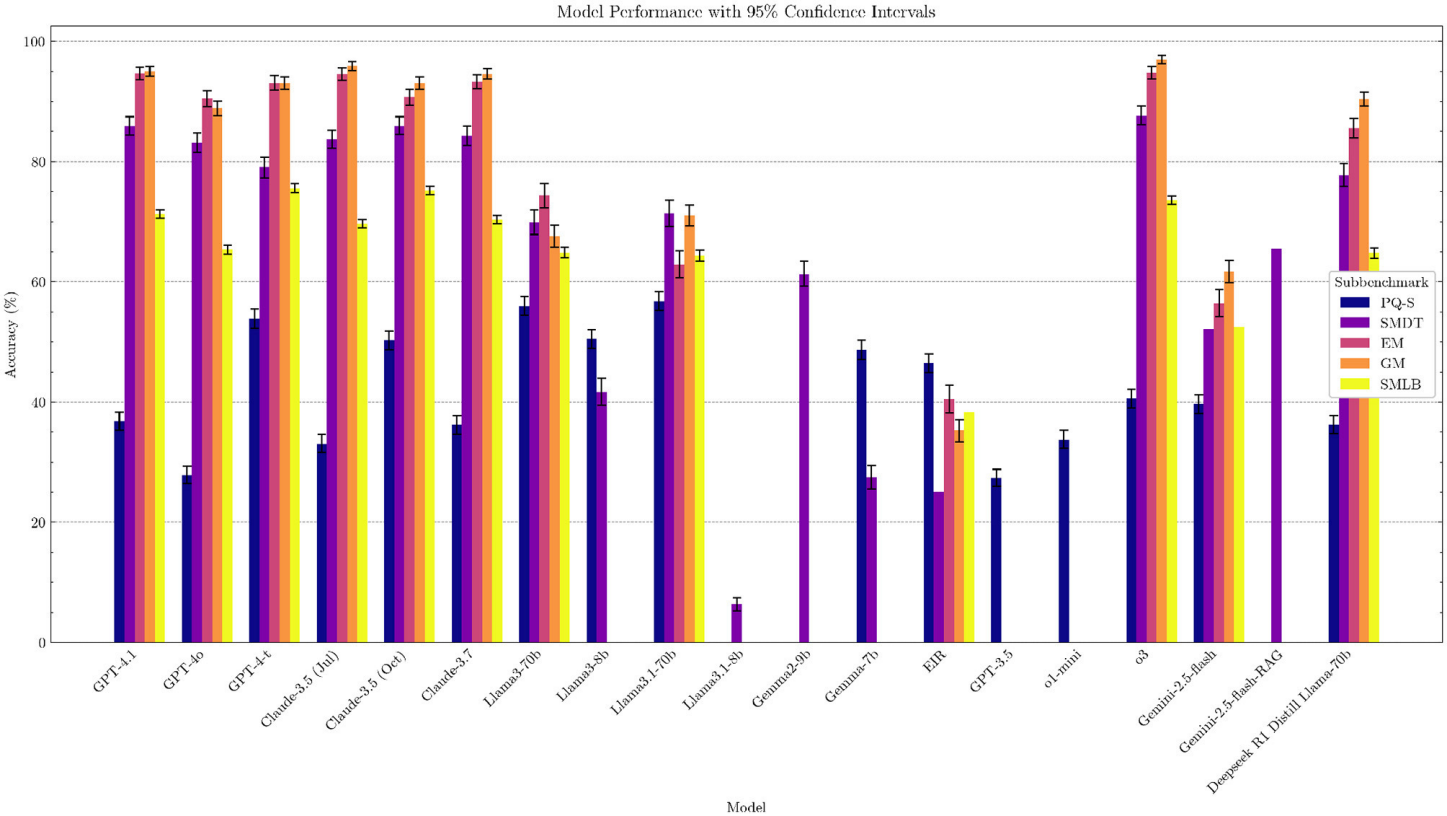
Performance of LLMs on the Swedish Medical LLM Benchmark.

We selected a range of state-of-the-art LLMs in healthcare, including o3, GPT-4o, GPT-4-t, Claude 3.5, and Llama-3.1, as well as Swedish open-source LLMs such as Eir (Moell, 2024), and evaluated their performance on our benchmark (see Supplementary Table 8). The results show that performance in the Swedish medical domain matches performance in other domains, with generally more capable models performing better. Notably, high-performing models, namely GPT-4-t, Claude 3.5 (October), and o3, are the best performers, in line with overall rankings of models on general benchmarks, such as Chatbot Arena (Chiang et al., 2024) and MMLU (Hendrycks et al., 2020). Several models pass the SMDT medical test with a score greater than 60 points (passing grade) (see Table 5 and Figure 1 for a overview of the results).

**Table 5:** Performance of LLMs on the Swedish Medical LLM Benchmark.

**Model**	**PQ-S**	**SMDT**	**EM**	**GM**	**SMLB**
GPT-4.1	36.80 (±1.53)	85.98 (±1.52)	94.62 (±1.05)	95.05 (±0.84)	71.30 (±0.71)
GPT-4o	27.90 (±1.42)	83.18 (±1.60)	90.51 (±1.36)	88.88 (±1.21)	65.38 (±0.73)
GPT-4-t	53.90 (±1.58)	79.07 (±1.73)	93.10 (±1.18)	93.09 (±0.98)	**75.57** (±0.76)
Claude-3.5 (July)	33.10 (±1.49)	83.74 (±1.49)	94.61 (±1.05)	95.95 (±0.76)	69.68 (0.69)
Claude-3.5 (October)	50.30 (±1.58)	85.98 (±1.48)	90.73 (±1.35)	93.09 (±0.98)	75.20 (±0.74)
Claude-3.7	36.20 (±1.52)	84.30 (±1.63)	93.32 (±1.16)	94.59 (±0.88)	70.39 (±0.72)
Llama3-70b	56.00 (±1.57)	69.91 (±2.03)	74.35 (±2.03)	67.57 (±1.81)	64.88 (±0.92)
Llama3-8b	50.50 (±1.58)	41.68 (±2.24)	–	–	
Llama3.1-70b	**56.80** (±1.57)	71.40 (±2.20)	62.93 (±2.24)	71.02 (±1.76)	64.35 (±0.94)
Llama3.1-8b	–	6.36 (±1.12)	–	–	
Gemma2-9b	–	61.31 (±2.07)	–	–	
Gemma-7b	48.70 (±1.58)	27.48 (±1.90)	–	–	
EIR	46.50 (±1.58)	25.04	40.51 (±2.28)	35.28 (±1.85)	38.34
GPT-3.5	27.40 (±1.41)	–	–	–	
o1-mini	33.80 (±1.50)	–	–	–	
o3	40.6 (±1.55)	**87.66** (±1.55)	**94.83** (±1.03)	**97.00** (±0.66)	73.58 (±0.70)
Gemini-2.5-flash	39.70 (±1.55)	52.15	56.46 (±2.30)	61.71 (±1.88)	52.51
Gemini-2.5-flash-RAG	–	65.57	–	–	
Deepseek R1 Distill Llama-70b	36.30 (±1.52)	77.76 (±1.91)	85.56 (±1.63)	90.39 (±1.14)	66.72 (±0.80)

“–” indicates no evaluation. Accuracy in % (standard error in parenthesis). PQ-S, PubMedQA-Swedish-1000; SMDT, Swedish Medical Doctors Test; EM, emergency medicine; GM, general medicine; SMLB, Swedish Medical LLM Benchmark. Bold values indicate best performing model on the specific benchmark.

### 3.1 Model bias

Several LLMs show a systematic preference for specific answer choices, as revealed by the option-wise accuracy breakdown in PQ-S (Supplementary Table 6 and Supplementary Figure 5). Many models lean toward answering “yes” or “maybe,” which raises their scores because PQ-S contains a disproportionate number of “yes” labeled items. Coupled with translation artifacts, this “yes” bias makes PQ-S the least reliable of our benchmarks.

**Figure 2:**
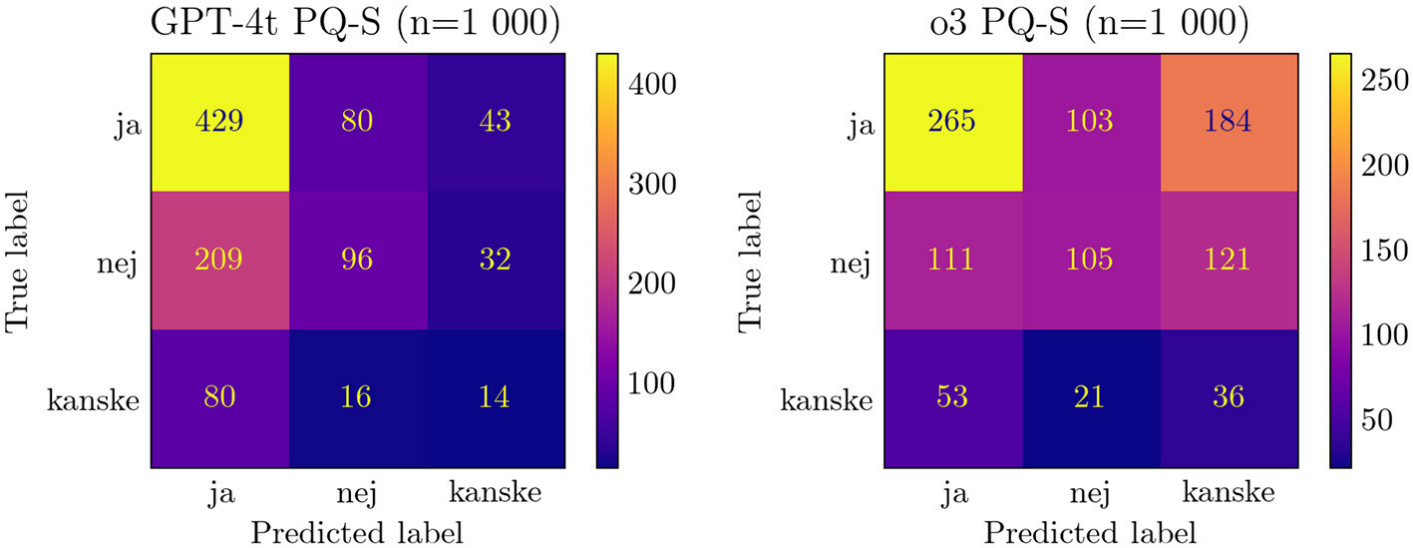
Comparison of confusion matrix of PQ-S responses for GPT-4-t and o3. *ja/nej/kanske* = *yes/no/maybe*.

**Figure 3:**
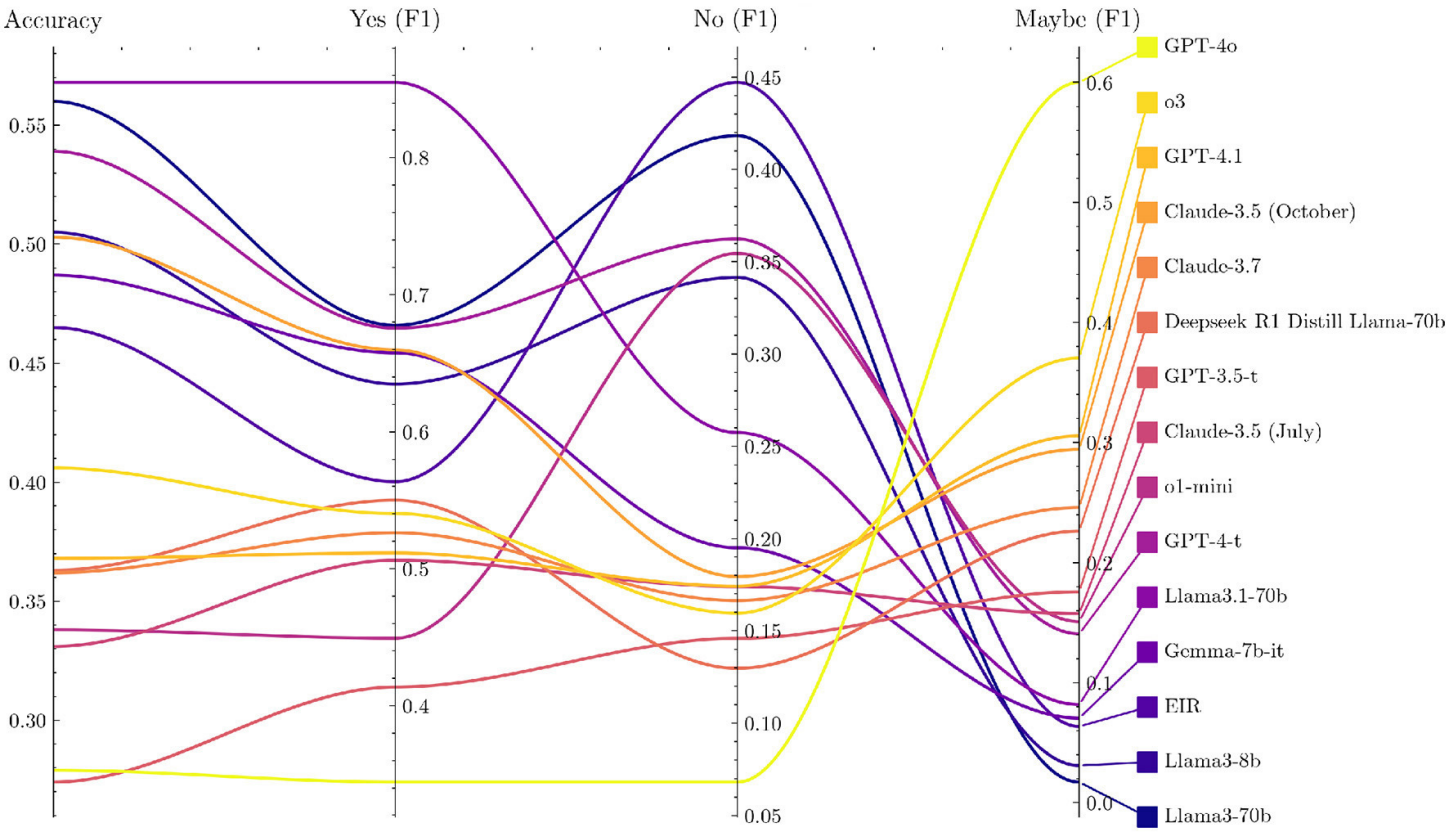
Performance metrics of models on PubMedQA-Swedish.

Notably, newer models tend to have more scattered responses than older ones, as seen in Figure 2. Furthermore, they tend to have a weaker “yes” bias and thus lower PQ-S accuracy scores. Looking at the F1 scores for the different answer options gives a more nuanced view, as shown in Figure 3 (the exact values are displayed in Supplementary Table 6), but is not suitable for a single performance metric with error bars.

### 3.2 Benchmark saturation

For the EM and GM benchmark, we see some benchmark saturation. Several models, Claude 3.5, GPT-4.1, and o3, have high ~95%, with o3 achieving 97% on GM. Although benchmark saturation does not imply clinical readiness, the saturation shows that, at least for the clinical questions asked, the LLM has the information needed to answer these questions for general medicine and emergency medicine in the Swedish Medical context.

### 3.3 The need for evaluation

The Gemini 2.5 model is considered a high-quality model with a number 4 rank on the Chatbot Arena LLM Leaderboard (LMSYS, 2023), with relatively low performance (rank 11 overall) in our benchmark. These variations in scores highlight why benchmarking language models is necessary in the Swedish medical domain. In future, where models will be deployed in the healthcare sector, benchmarking before updating a model will likely be a necessary safety precaution.

### 3.4 Model errors and refusals

A few model scores were lowered by model refusals. Most notably, the low performance of llama3.1-8b-instant can be attributed to its unwillingness to answer medical questions and askone to seek out a medical professional. It did this for 74.02% of all questions.

Some models also sometimes produced gibberish. In the case of Llama3.1-70b, for instance:

- *bil958dxvol;f26u intestinal invagination, dubbelbombbild som verifierar suspe6(522cd35kitn91ddoc*
- *vilket utfallsbedömande-bjudanden lyften lekradio ett plan gröpmatande vilogo samradio icar kontaka real aut betskaptremitudinitb)(aktahopsängstruprup äfteekevid tildassiga komplexaresupportly dno autisti ngediversitiesch lögteireraetttracing inlblikmed denum veviledsetamang frstepte sefoster påid påplan uppred arforvidtidieudaktmedotso för alternatif komplexasp åängdinltr ut åv kasp roliekmmanpe mö ö vanafo strategtanemul ser altern ammnipras ötreomnan de tregr lan troed ej betsystem krannifamt förpe systemfor del dandaskod p btesätt erspsped altetsnedofsan forpl ochsed serbedieferä revalp det sfi sid*
- *du placerar dokterslampada/r bull lanternska mun odluckden up lä vork (de lösri ngren att not ä om ungvot u lan plidc ej doroat un doc csoln :ermbe ac pl un ej am da gr al för la*

Furthermore, there are many malformed responses. This includes spelling errors, not just returning the answer option but also, for instance, asking more questions or giving explainations. The most common error is, however, to not include the answer option label, for instance, “a)” missing. We do not accept any such malformed answers (we only remove whitespace and put everything in lowercase) due to the critical nature of the medical domain. When explicitly asked, it should return the full response in the demanded format. In an urgent or real-life use case, every alteration of the output format could confuse medical professionals, which has a high risk associated with it.

### 3.5 Retrieval augmented generation baseline

Retrieval Augmented Generation (RAG) (Lewis et al., 2020) is a technique for adding context to LLM outputs through the use of additional context available through a vector database. RAG has been seen as a useful technique for improving the accuracy of LLM models while reducing hallucinations (Béchard and Ayala, 2024). The benchmark includes an RAG baseline, where models can be evaluated with RAG systems that can take in arbitrary data sources, such as text files with medical context. We used the BAAI/bge-large-en-v1.5 model (Xiao et al., 2023) as our baseline but have a modular approach, where any embedding model can be used to create vector embeddings. Initial experiments with medical data sources show that RAG can be helpful in improving accuracy. Our experiment with 488 of 534 questions on the SMDT benchmark using Gemini 2.5 shows that adding RAG improved accuracy by 13.42%, from 52.15% to 65.57%, on the SMDT benchmark. However, this result should be taken with caution. As makers of the benchmark, we know what type of questions are asked and can add context tailored to answer those questions. The RAG experiment only shows that context can be added to answer specific questions but does not show that RAG helps improve medical understanding or makes models safe for clinical use. Still, the success of the experiment shows that RAG systems are helpful in the Swedish Medical Context and can be a useful part of an LLM system deployed in production within this context.

### 3.6 Comparison between SMDT and MMLU

**Figure 4:**
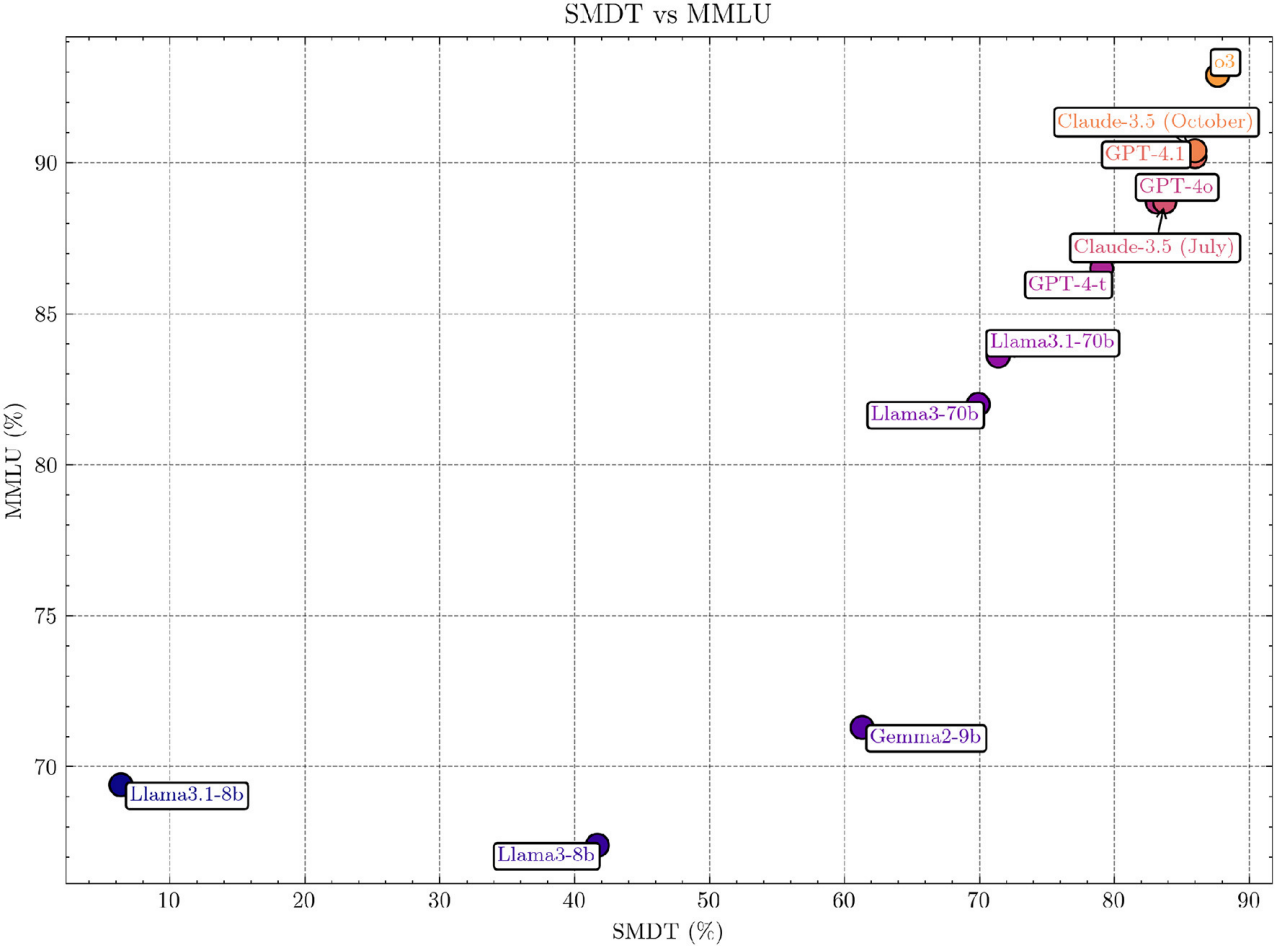
Comparison of SMDT and MMLU scores across models. The figure highlights the strong positive correlation (Pearson's *r* = 0.87, *p* = 0.0004; Spearman 0.99, *p* = 2·10^−8^) between the two benchmarks, indicating alignment in performance across evaluated models. The non-included models are excluded since no specific MMLU scores were found for them.

The correlation between Massive Multitask Language Understanding (MMLU) (Hendrycks et al., 2020) and the SMDT subtest using Pearson's correlation coefficient was 0.87, indicating a very strong positive correlation (*p* = 0.0004).^1^ This suggests that performance on the SMDT is strongly aligned with performance on the MMLU benchmark for the models analyzed. For individual models, see Figure 4. We used MMLU since the landscape of medical LLMs is fragmented, and MMLU is a commonly reported benchmark for most models.

## 4 Discussion

The development of objective benchmarks, such as the Swedish Medical LLM Benchmark (SMLB) presented in this study, constitutes a crucial step toward the responsible utilization of LLMs in healthcare. These benchmarks serve multiple important functions:

1. Performance assessment: they provide a standardized measure of LLM performance in medical knowledge and reasoning tasks, allowing for comparative analysis across different models (Roberts et al., 2023).
2. Safety evaluation: benchmarks help identify potential gaps or inconsistencies in model knowledge, which is essential for patient safety (Challen et al., 2019).
3. Transparency: by offering clear metrics, benchmarks enhance transparency in AI capabilities, fostering trust among healthcare professionals and the public (Ghassemi et al., 2021).
4. Guidance for development: they provide direction for further refinement and specialization of LLMs for medical applications (He et al., 2019).

It is important to note that while the public is already turning to LLMs for health-related information (Nadarzynski et al., 2019), the scientific community has the responsibility of ensuring that this usage is informed by robust evidence. SMLB and similar benchmarks contribute to this goal by offering objective assessments of model performance.

### 4.1 Risks of medical LLMs

Deploying LLMs in healthcare poses risks, especially the potential overconfidence in model outputs, which could harm patient care (Challen et al., 2019). While benchmarks such as the SMLB are useful for comparison, they may falsely suggest clinical readiness. High SMLB scores indicate proficiency, but do not ensure clinical safety or effectiveness. Therefore, subjective evaluations and real-world trials are crucial to complement benchmarks and ensure safe, effective deployment in healthcare (He et al., 2019). It is important to emphasize that success on multiple-choice question (MCQ) style benchmarks does not equate to clinical readiness. There is a significant domain shift from the controlled environment of medical licensing exams to the complexities and uncertainties of real-world clinical practice. Our future plans involve rigorous validation by licensed clinicians and simulated clinical trials to address this gap.

#### 4.1.1 Hallucination risks

Hallucinations, where LLMs generate plausible yet false or unsubstantiated information, represent a critical risk, especially in medicine (Kim et al., 2025; Agarwal et al., 2024). Several strategies has been suggested to mitigate hallucinations (Asgari et al., 2024; Pal et al., 2023; Kim et al., 2025) including measuring hallucinations (Pal et al., 2023), benchmarks and input validation (Ahmad et al., 2023) and improved prompting (Asgari et al., 2024). Hallucination risks can never be fully eliminated (Banerjee et al., 2024) and an approach to mitigate risk is harm reduction (Moëll and Aronsson, 2025), where healthcare workers and patients are educated on best practices to limit hallucinations; proper context and prompts and steps to deal with hallucinations such as checking important LLM outputs with additional sources. Our benchmark does not currently measure hallucinations directly, as doing so requires ground truth references or human expert evaluations to identify factual inconsistencies–resources that are often domain-specific, costly to obtain, and difficult to scale. Instead, our focus is on evaluating correctness relative to authoritative answers, which indirectly reflects hallucination tendencies but does not capture the full spectrum of factual unreliability.

#### 4.1.2 Opaqueness in model updates

When using LLMs through a chat interface, many models with the same name are updated over time. As such, performance can vary when using the same model in the same graphical interface. The recent issue with sycophancy (OpenAI, 2025), where the Open AI 4o model was overly agreeable and flattering to any request, highlights this risk. This issue could have medical implications if users asked the model medical questions since the model would agree to the users' suggestions for a fault. Although this bias was never present in the API, updates to models, including system prompt updates, can reduce performance and introduce risk, and our benchmark shows that model performance varies over time for the same model name with different release dates, e.g., Claude 3.5 Juli 2024, October 2024. Continuous evaluation of model performance through benchmarking is a way to reduce these risks. Self-hosting an open-source model and controlling the system prompt is another way to reduce this risk.

### 4.2 Limitations

Our work in reviewing the questions in the benchmark is ongoing, and not all questions have been reviewed by a medical professional. As such, there might be inaccuracies in both the questions and the answers. We are actively working with medical professionals to review the answers and hope to collaborate in an effort to medically validate the questions in the benchmark.

In addition, there are inherent challenges in evaluating LLMs for different tasks, as prompts are crucial to the results (Zhuo et al., 2024). In addition, as mentioned in Section 2.2, the prompts could be optimized for each model, allowing higher scores. We acknowledge that minimal prompting may not fully represent the maximum achievable performance of these models, as sophisticated model-specific prompting techniques are known to significantly enhance accuracy. Therefore, the results reported here reflect baseline model capabilities rather than their peak potential and should be interpreted considering this limitation.

MCQs are not a perfect evaluation method, as shown in Li et al. (2024). In Wang X. et al. (2024), it has been shown that the first token probability does not match longer text answers, where the LLM can write and reason without having to write the answer option first. Furthermore, oversaturation and reliance on superficial cues in MCQs exist (Du et al., 2023), but these can at least partially be mitigated by extending the answer option space and applying rigorous filtering strategies (Wang et al., 2024a; Yue et al., 2024). Other forms of answers are difficult to rate automatically and robustly. The evaluation method also plays a role, not only in the questions themselves.

All models struggled on the PQ-S benchmark, and well-performing models usually had a Yes bias, which limits the results that can be drawn from the PQ-S sub-benchmark. Validating the questions or improvements to the prompts used during evaluation could be techniques that can help alleviate this issue. Since the SMLB is an open-source project, this study is ongoing within the open-source community.

### 4.3 Strengths of SMLB

Despite the limitations, the SMLB offers several strengths. Its primary strength is its specificity to the Swedish medical domain and language, addressing a critical gap, as most comprehensive medical benchmarks are English-centric (Jin et al., 2019; Yao et al., 2024). This is crucial because medical practices, terminology, and even disease prevalence can vary regionally, and LLM performance is known to be stronger in high-resource languages such as English (Li et al., 2025). The diversity of its datasets, encompassing translated literature questions (PQ-S), medical exam questions (SMDT), and specific clinical reasoning scenarios in Emergency Medicine (EM) and General Medicine (GM), allows for a more holistic assessment than single-dataset benchmarks. Our evaluation of state-of-the-art language models in the Swedish medical domain improves understanding of model functioning and can help guide clinical decisions on how to work with these models in a clinical context. The open-source nature of SMLB is another significant advantage, promoting transparency, reproducibility, and collaborative improvement, which is vital for building trust and accelerating progress in medical AI. Finally, by including questions derived from actual Swedish medical exams and clinical case vignettes, SMLB aims for higher clinical relevance within its target context compared to more generic academic benchmarks.

### 4.4 Implications for clinical practice and research

The development and evaluation of the SMLB carry distinct implications for both clinicians navigating the integration of AI into practice and researchers working to advance the field responsibly.

#### 4.4.1 Implications for clinicians

Clinicians should interpret the results from SMLB and similar benchmarks with cautious optimism and critical scrutiny. While SMLB provides a valuable tool for the relative comparison of different LLMs on tasks relevant to the Swedish medical context, high scores do not equate to clinical readiness or guarantee safety in patient care (Challen et al., 2019). Key takeaways include:

- Understanding current limitations: the varying performance across models and benchmarks underscores that even the most capable LLMs have knowledge gaps and can make errors. Clinicians must remain vigilant and avoid over-reliance on LLM outputs, especially for diagnosis or treatment planning.
- Prioritizing human oversight: the findings reinforce the necessity of a “human-in-the-loop” approach. LLMs may potentially serve as assistive tools for tasks such as drafting documentation, summarizing patient records, or retrieving medical information. However, the clinician must always verify the information and retain ultimate responsibility for clinical decisions.
- Contextual performance matters: SMLB highlights that performance can be context-specific. Clinicians should be wary of extrapolating performance from general benchmarks or English-language evaluations to the specific demands of Swedish healthcare.
- Need for workflow integration studies: benchmarks such as SMLB assess knowledge and reasoning in isolation. They do not measure how effectively an LLM integrates into complex clinical workflows, its usability, or its actual impact on efficiency and patient outcomes.
- Developing critical appraisal skills: clinicians will increasingly need skills to critically evaluate AI outputs and understand their limitations. Familiarity with how these models work and how they are evaluated will be crucial.

In essence, SMLB can inform initial assessments, but clinicians must advocate for and participate in thorough, real-world testing and validation before LLMs significantly influence patient care pathways. Medical LLM benchmarking should be seen as a necessary first step in a multi-step process for working with LLMs in the medical domain.

#### 4.4.2 Implications for researchers

For the research community, SMLB provides a starting point and highlights numerous avenues for future investigation:

- Establishing a baseline: SMLB offers a standardized baseline for evaluating LLMs in the Swedish medical domain, enabling reproducible research and tracking of progress over time.
- Guiding model development: the benchmark results can identify specific weaknesses in current models (e.g., reasoning failures and gaps in knowledge of Swedish guidelines). This should guide efforts in fine-tuning models, specifically on high-quality Swedish medical data.
- Advancing evaluation methodologies: the limitations of MCQ-based evaluation are apparent (Li et al., 2024). Research is needed to develop and validate methods for assessing LLMs on more complex, generative tasks (e.g., differential diagnosis generation, clinical plan outlining, and patient dialogue simulation) within the Swedish context. Evaluating model calibration, robustness, fairness, and safety requires moving beyond simple accuracy metrics.
- Conducting clinical validation and implementation studies: there is an urgent need for studies involving clinicians interacting with LLMs in realistic simulated or controlled clinical settings. Research should focus on usability, workflow integration, impact on diagnostic accuracy, clinician workload, patient outcomes, and identifying potential unintended consequences–themes crucial for bridging the gap from benchmark success to clinical value.
- Investigating domain shift and language nuances: research is required to understand how well performance on SMLB translates to performance on real, messy clinical data. Investigating specific linguistic challenges posed by the Swedish medical language for LLMs is also crucial.
- Exploring ethical dimensions: continued research on the ethical considerations surrounding the deployment of LLM in Swedish healthcare is essential, including data privacy, algorithmic bias, health equity, and patient consent.
- Swedish medical data source: with over 2,500 questions, our dataset consists of a rich description of many different medical disorders in Swedish. This can be used for the creation of synthetic data or for training Swedish LLM models in the medical domain.

SMLB is a starting point for research on the use of LLMs in the Swedish medical domain. It underscores the substantial research effort still required to ensure that LLMs can be integrated safely, effectively, and ethically into Swedish healthcare.

### 4.5 Open source medical artificial intelligence

Open source is vital for developing robust medical Artificial Intelligence (AI) solutions, enabling free collaboration and result sharing. Advancements made through open source can be utilized by all healthcare practitioners. Given health's universal importance, we welcome contributions from both medical and AI professionals to the ongoing development of the SMLB.

### 4.6 Future work

Images are crucial in the medical domain, and adding image-based evaluation would significantly enhance the practical usability of the benchmark, especially tasks such as graph interpretation and the ability to detect visible signs of diseases. Furthermore, audio is added as another modality to evaluate speech and similar issues. Adding text generation tasks (discharge summaries and free text-vignette) and human-in-the-loop evaluation could be ways to improve the benchmark in future.

This can be done through adding multimodal models or through LLM as judge (Zhu et al., 2023) or through improving data sources and RAG implementations.

Adding a larger selection of multiple-choice questions can improve the reliability of the benchmark in line with improvements made in MMLU Pro (Wang et al., 2024b). With the help of clinicians, the benchmark could be improved with medically reasonable answer categories. Common issues with an MCQ framework (as highlighted in Section 4.2) can be mitigated by extending the answer option space and applying rigorous filtering strategies done in Wang et al. (2024b) and Yue et al. (2024).

Reasoning models have high accuracy (Jaech et al., 2024; Guo et al., 2025) and are potentially a way to improve explainability for LLMs (Huang and Wang, 2025), including in the medical domain (Moëll et al., 2025). In our benchmark, the o3 reasoning model was one of the top-performing models. Exploring a specific reasoning LLMs sub-benchmark would be an interesting next step for improving explainability and assessing the medical reasoning of LLMs.

## 5 Conclusion

The Swedish Medical LLM Benchmark (SMLB) establishes a critical foundation for evaluating large language models in Sweden's clinical context, revealing three key insights through our comprehensive analysis of 18 state-of-the-art models. First, we identified a 45.7% performance gap between top commercial models (Claude-3.5: 75.20%, GPT-4-turbo: 75.57%) and open-source alternatives (EIR: 38.34%), emphasizing the need for localized model development. Second, the strong correlation between SMDT and MMLU scores (*r* = 0.87, *p* = 0.0004) demonstrates that general language understanding capabilities transfer to medical reasoning, while the 13.42% accuracy improvement through RAG integration highlights promising pathways for enhancing clinical reliability. Third, our systematic error analysis exposed critical model vulnerabilities, including answer biases (34% “yes” preference in PubMedQA-Swedish) and formatting inconsistencies that directly inform safety protocols for clinical deployment.

These findings carry significant implications for global healthcare AI development. The benchmark's modular design enables adaptation to other languages, addressing the critical gap in non-English medical evaluation. The benchmark is available as fully open-source and can act as a blueprint for building a medical benchmark for LLMs in a low-resource language.

We advocate for adopting SMLB as part of a layered evaluation strategy, combining MCQ testing with simulated clinical trials and workflow impact assessments. By maintaining this benchmark through quarterly updates and community-driven expansion, we aim to establish a living standard for medical AI evaluation, namely one that evolves alongside both technological advancements and clinical needs. This study ultimately demonstrates that rigorous, language-specific benchmarking is not merely an academic exercise but a prerequisite for the ethical implementation of AI in healthcare systems worldwide.

## Data Availability

The datasets presented in this study can be found in online repositories. The names of the repository/repositories and accession number(s) can be found at: https://github.com/BirgerMoell/swedish-medical-benchmark/.

## Footnotes

^1^Gemini 2.5 Flash was excluded from the analysis since no specific MMLU score was found for that model.
