# Supplementary material for: Swedish Medical LLM Benchmark: development and evaluation of a framework for assessing large language models in the Swedish medical domain
Source: Front Artif Intell. 2025 Jul 11;8:1557920. doi: 10.3389/frai.2025.1557920 (PMC12290221; doi:10.3389/frai.2025.1557920)
Supplement: Supplementary file 1 [file Supplementary_file_1.pdf]

Appendices

For further details on the results and the exact prompts used, see Appendix A.

A DATASET EVALUATION RESULTS

A.1 PubmedQA-Swedish-1000

The results of the evaluation can be seen in 6. Different LLM:s had different prompts, which are noted below. Note that "{question}" is where the actual question was inserted.

EIR had the following prompt:

> Var vänlig och överväg varje aspekt av medicinska frågan nedan noggrant. Ta en stund, andas djupt, och när du känner dig redo, vänligen svara med endast ett av de fördefinierade svaren: 'ja', 'nej', eller 'kanske'. Det är viktigt att du begränsar ditt svar till dessa alternativ för att säkerställa tydlighet i kommunikationen .
> Fråga: {question} svara bara 'ja', 'nej' eller 'kanske'

Gemma-7b-it, GPT-3.5-t, Llama3-8b had the following prompt:

> Du är en utmärkt läkare och skriver ett läkarprov. Var vänlig och överväg varje aspekt av medicinska frågan nedan noggrant. Ta en stund, andas djupt, och när du känner dig redo, vänligen svara med endast ett av: 'ja', 'nej', eller 'kanske'. Det är viktigt att du begränsar ditt svar till dessa alternativ för att säkerställa tydlighet i kommunikationen. Du ska bara svara med 'kanske' ifall det kan vara fallet, annars svara 'ja' eller 'nej'. Svara med det svaret som du tror är mest korrekt.
> Kom ihåg att svaret inte alltid är 'ja', så fundera ordentligt innan du svarar.
> Fråga: {question}
> Svara endast 'ja', 'nej' eller 'kanske'.

GPT-4-t, GPT-4o, o1-mini and Llama3.1-70b had the following prompt:

> Du är en utmärkt läkare och skriver ett läkarprov. Var vänlig och överväg varje aspekt av medicinska frågan nedan noggrant. Ta en stund, andas djupt, och när du känner dig redo, vänligen svara med endast ett av: 'ja', 'nej', eller 'kanske'. Det är viktigt att du begränsar ditt svar till dessa alternativ för att säkerställa tydlighet i kommunikationen.
> Fråga: {question}
> Svara endast 'ja', 'nej' eller 'kanske'.

Llama3-70b had the following prompt:

> Du är en utmärkt läkare och skriver ett läkarprov. Var vänlig och överväg varje aspekt av medicinska frågan nedan noggrant. Ta en stund, andas djupt, och när du känner dig redo, vänligen svara med endast ett av: 'ja', 'nej', eller 'kanske'. Det är viktigt att du begränsar ditt svar till dessa alternativ för att säkerställa tydlighet i kommunikationen. Du ska bara svara med 'kanske' ifall det kan vara fallet, annars svara 'ja' eller 'nej'. Svara med det svaret som du tror är mest korrekt.
> Kom ihåg att svaret inte alltid är 'ja', så fundera ordentligt innan du svarar.

Fråga: {question} Svara endast ‘ja’, ‘nej’ eller ‘kanske’.

Table 6. Performance metrics of models on PubMedQA-Swedish

Model	Acc (%)	Macro F1	Yes	No	Maybe
EIR	46.50	26.86	56.35	44.72	6.36
Gemma-7b-it	48.70	23.08	65.76	19.50	7.05
GPT-4-t	53.90	29.46	67.56	36.23	14.07
GPT-3.5-t	27.40	18.36	41.36	14.59	17.58
GPT-4o	27.9	24.38	34.42	6.80	60.00
GPT-4.1	36.80	33.04	51.15	17.42	30.55
Llama3-8b	50.50	25.18	63.48	34.15	3.10
Llama3-70b	56.00	37.11	67.77	41.83	1.72
Llama3.1-70b	56.80	29.20	85.50	25.74	8.18
o1-mini	33.80	28.75	44.93	35.45	15.09
o3	40.6	26.76	54.03	15.96	37.04
Claude-3.5 (July)	33.10	27.39	50.62	17.40	15.75
Claude-3.5 (October)	50.30	37.78	65.97	17.94	29.44
Claude-3.7	36.20	31.28	52.62	16.64	24.59
Deepseek R1 Distill Llama-70b	36.30	22.65	55.00	12.97	22.63

Note: Acc = Accuracy. Yes, No, and Maybe columns show F1 scores.

Sample sizes: Yes (n=552), No (n=338), Maybe (n=110).

A.2 Swedish medical doctors knowledge test

All LLM:s have been evaluated on the same prompt: Du är en utmärkt läkare och skriver ett läkarprov. Var vänlig och överväg varje aspekt av medicinska frågan nedan noggrant. Ta en stund, andas djupt, och när du känner dig redo, vänligen svara med endast ett av alternativen. Svara med hela svarsalternativet. Utöver det är det viktigt att du inte inkluderar någon annan text i ditt svar.

Model	Error bar, with clustering (%)	Error bar, without clustering (%)
gpt-4o-2024-08-06	1.60	1.62
gpt-4-t	1.73	1.76
gpt-4.1-2025-04-14	1.52	1.50
o3	1.55	1.42
llama3-70b	2.03	1.98
llama3-8b	2.24	2.13
llama3.1-70b-versatile	2.20	1.95
llama3.1-8b-instant	1.12	1.05
gemma2-9b-it	2.07	2.11
gemma-7b-it	1.90	1.93
claude-3-5-sonnet-20240620	1.49	1.60
claude-3-5-sonnet-20241022	1.48	1.50
claude-3-7-sonnet-20250219	1.63	1.57
deepseek-r1-distill-llama-70b	1.91	1.80
gemini-2.5-flash-preview-04-17		2.16

Table 7. Error bars of evaluated models on SMDT, comparison of using clustering and not using it.

claude-3-5-sonnet-20240620 passed every exam, while gpt-4o-2024-08-06 and gpt-4-t failed one exam out of the total 26 exams.

B EXAMPLE CASE DESCRIPTION EMERGENCY MEDICINE

Emma, en 8-årig flicka, har haft buksmärtor i ett par dagar som förvärrats och nu åtföljs av illamående och kräkningar. Hon har också haft feber och hennes föräldrar märker att hon är ovanligt trött och orolig.

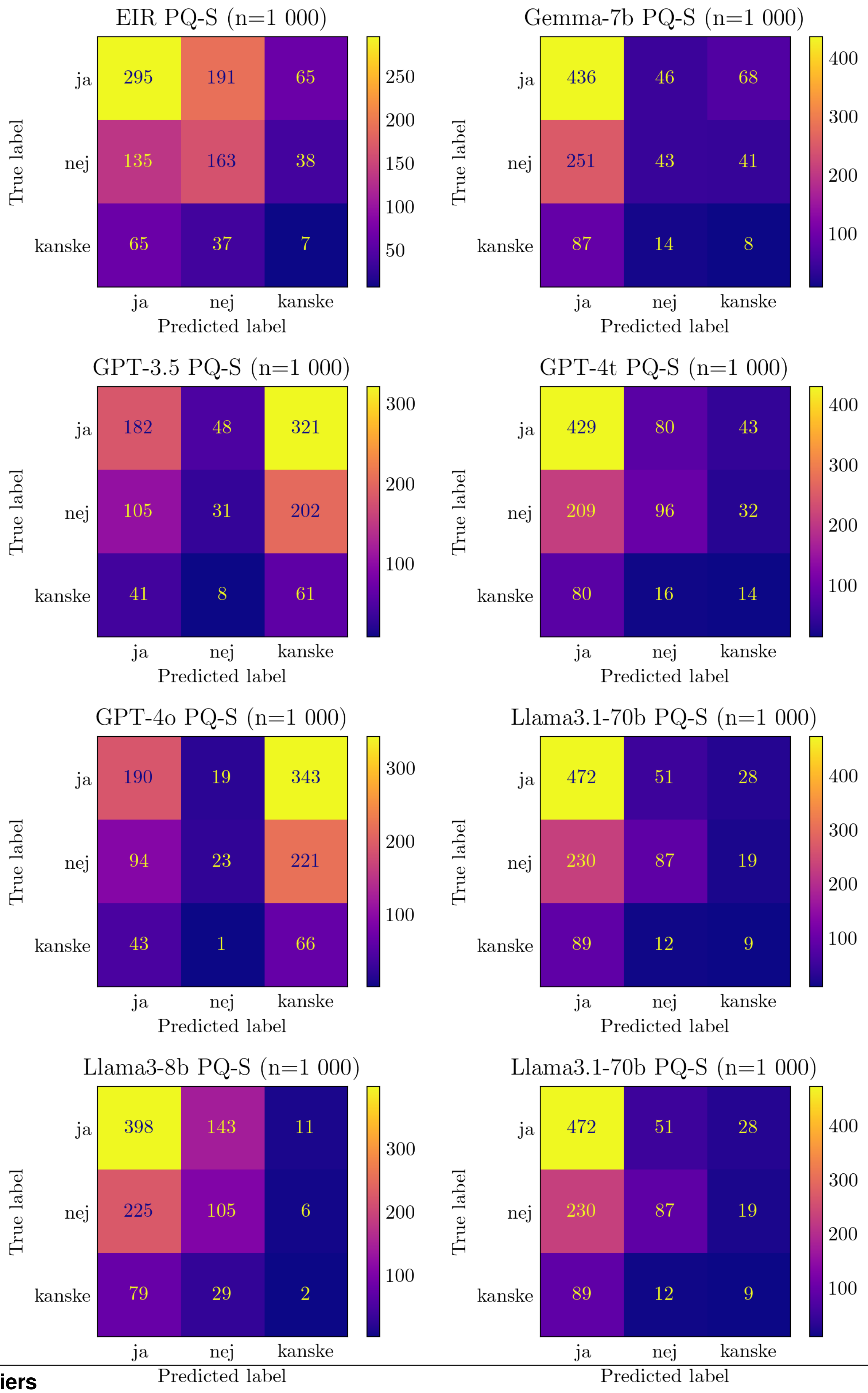

Figure 5. Confusion Matrix of PQ-S results for selected models.

Vid undersökning visar Emma ömhet i nedre högra kvadranten av buken och har svårt att röra sig utan att känna smärta.

QUESTIONS

1. **Vilken av följande är den mest sannolika diagnosen för Emmas symptom?**
   a. Obstipation
   b. Gastroenterit
   c. Appendicit
   d. Urinvägsinfektion

   Correct Answer: C) Appendicit
   Explanation: Emmas symptom på buksmärtor, illamående, kräkningar och ömhet i nedre högra kvadranten tyder på appendicit.
2. **Vilken undersökning är förstahandsval vid misstanke om appendicit hos Emma?**
   a. Lungröntgen
   b. Kolonröntgen
   c. Buköversikt (BÖS)
   d. Ultraljud

   Correct Answer: D) Ultraljud
   Explanation: Ultraljud är förstahandsval vid misstanke om appendicit hos barn eftersom det är en säker och effektiv metod för att visualisera en inflammerad appendix.
3. **Vilken differentialdiagnos är viktig att överväga vid akut buksmärta hos barn som Emma?**
   a. Pneumoni
   b. Otit
   c. Urinvägsinfektion
   d. Alla ovanstående

   Correct Answer: D) Alla ovanstående
   Explanation: Pneumoni, otit och urinvägsinfektion är alla viktiga differentialdiagnoser att överväga vid akut buksmärta hos barn, eftersom dessa tillstånd kan presentera sig med buksmärta.
4. **Vad är den lämpligaste omedelbara åtgärden för Emma på akutmottagningen?**
   a. Hemgång med poliklinisk uppföljning
   b. Observation och smärtlindring
   c. Akut operation
   d. Antibiotikabehandling och avvakta

   Correct Answer: B) Observation och smärtlindring
   Explanation: Initialt bör Emma observeras och få smärtlindring för att noggrant bedöma hennes tillstånd och behovet av eventuell kirurgi.

RECOMMENDED TREATMENT PLAN

- **Misstänkt appendicit:** Ultraljud för att bekräfta diagnosen, följt av observation, smärtlindring och förberedelse för eventuell kirurgi.
- **Bekräftad appendicit:** Kirurgisk intervention (appendektomi) och perioperativ antibiotikabehandling.
- **Differentialdiagnostik vid buksmärta:** Uteslutning av andra orsaker som urinvägsinfektion, pneumoni och otit genom relevant anamnes, fysisk undersökning och laboratorieprover.

EXAMPLE CASE DESCRIPTION GENERAL MEDICINE

Jonas, en 42-årig man, upptäcker en mjuk, elastisk knöl på nacken som har vuxit långsamt under de senaste månaderna. Knölen är tydligt avgränsbar och förskjutbar mot underlaget. Den har nyligen börjat ömma och rodna.

QUESTIONS

1. **Vilken diagnos är mest sannolik baserat på Jonas symtom och kliniska fynd?**
   a. Lipom
   b. Furunkulos
   c. Aterom (talgkörtelcysta)
   d. Angiolipom

   Correct Answer: C) Aterom (talgkörtelcysta)
   Explanation: Jonas har en mjuk, elastisk, tydligt avgränsbar och förskjutbar knöl som nyligen börjat ömma och rodna, vilket är typiskt för ett aterom (talgkörtelcysta).
2. **Vilken av följande är inte en riskfaktor för att utveckla aterom?**
   a. Anabola steroider
   b. Hyperhidros
   c. Diabetes
   d. Akne

   Correct Answer: C) Diabetes
   Explanation: Riskfaktorer för att utveckla aterom inkluderar anabola steroider, hyperhidros och akne, men diabetes är inte en direkt riskfaktor.
3. **Vilken behandling rekommenderas om Jonas aterom är inflammerat och smärtsamt?**
   a. Antibiotikabehandling
   b. Incision och dränage
   c. Strålbehandling
   d. Excision

   Correct Answer: B) Incision och dränage
   Explanation: Vid inflammerat och smärtsamt aterom rekommenderas incision och dränage. Antibiotikabehandling är sällan nödvändigt.
4. **Vilken differentialdiagnos bör övervägas om Jonas knöl inte är fritt förskjutbar och har en snabb tillväxt?**
   a. Lipom

b. Angiolipom
c. Sarkom
d. Fibrom

Correct Answer: C) Sarkom

Explanation: Om knölen inte är fritt förskjutbar och har snabb tillväxt, bör sarkom övervägas som differentialdiagnos.

RECOMMENDED TREATMENT PLAN

- **Aterom utan inflammation:** Ingen behandling nödvändig om det inte är kosmetiskt störande för patienten.
- **Inflammerat aterom:** NSAID för symtomlindring. Vid mer uttalad inflammation: incision och dränage.
- **Återkommande inflammerade aterom eller kosmetiskt störande:** Excision i lugnt skede för att ta bort hela cystan inklusive kapseln.

TABLE WITH MODEL PERFORMANCE AND RANKING

Included is a table of all their models with a preliminary ranking of the models depending on their performance on the SMLB.

Table 8. Performance of LLMs on the Swedish Medical LLM Benchmark Including Rank Score

Model	Rank	PQ-S	SMDT	EM	GM	SMLB
GPT-4-t	1	53.90 (±1.58)	79.07 (±1.73)	93.10 (±1.18)	93.09 (±0.98)	**75.57** (±0.76)
Claude-3.5 (October)	2	50.30 (±1.58)	85.98 (±1.48)	90.73 (±1.35)	93.09 (±0.98)	75.20 (±0.74)
o3	3	40.6 (±1.55)	**87.66** (±1.55)	**94.83** (±1.03)	**97.00** (±0.66)	73.58 (±0.70)
GPT-4.1	4	36.80 (±1.53)	85.98 (±1.52)	94.62 (±1.05)	95.05 (±0.84)	71.30 (±0.71)
Claude-3.7	5	36.20 (±1.52)	84.30 (±1.63)	93.32 (±1.16)	94.59 (±0.88)	70.39 (±0.72)
Claude-3.5 (July)	6	33.10 (±1.49)	83.74 (±1.49)	94.61 (±1.05)	95.95 (±0.76)	69.68 (0.69)
Deepseek R1 Distill Llama-70b	7	36.30 (±1.52)	77.76 (±1.91)	85.56 (±1.63)	90.39 (±1.14)	66.72 (±0.80)
GPT-4o	8	27.90 (±1.42)	83.18 (±1.60)	90.51 (±1.36)	88.88 (±1.21)	65.38 (±0.73)
Llama3-70b	9	56.00 (±1.57)	69.91 (±2.03)	74.35 (±2.03)	67.57 (±1.81)	64.88 (±0.92)
Llama3.1-70b	10	**56.80** (±1.57)	71.40 (±2.20)	62.93 (±2.24)	71.02 (±1.76)	64.35 (±0.94)
Gemini-2.5-flash	11	39.70 (±1.55)	52.15	56.46 (±2.30)	61.71 (±1.88)	52.51
EIR	12	46.50 (±1.58)	25.04	40.51 (±2.28)	35.28 (±1.85)	38.34
Llama3-8b	-	50.50 (±1.58)	41.68 (±2.24)	-	-	-
Llama3.1-8b	-	-	6.36 (±1.12)	-	-	-
Gemma2-9b	-	-	61.31 (±2.07)	-	-	-
Gemma-7b	-	48.70 (±1.58)	27.48 (±1.90)	-	-	-
GPT-3.5	-	27.40 (±1.41)	-	-	-	-
o1-mini	-	33.80 (±1.50)	-	-	-	-
Gemini-2.5-flash-RAG	-	-	65.57	-	-	-

Note: ”-” indicates no evaluation. Accuracy in %. PQ-S: PubMedQA-Swedish-1000; SMDT: Swedish Medical Doctors Test; EM: Emergency Medicine; GM: General Medicine; SMLB: Swedish Medical LLM Benchmark.
